# The emergence of insecticide resistance in the major malaria vector *Anopheles funestus* (Diptera: Culicidae) from sentinel sites in Mutare and Mutasa Districts, Zimbabwe

**DOI:** 10.1186/s12936-015-0993-8

**Published:** 2015-11-20

**Authors:** Shadreck Sande, Moses Zimba, Peter Chinwada, Hieronymo Takudzwa Masendu, Sungai Mazando, Aramu Makuwaza

**Affiliations:** Department of Biological Science, University of Zimbabwe, Harare, Zimbabwe; Abt Associates Inc., 1 Erskine Road, Mt Pleasant, Harare, Zimbabwe; National Institute of Health Research, Causeway, Harare, Zimbabwe

**Keywords:** *Anopheles funestus*, Insecticide resistance, Malaria vectors, Indoor residual spraying, Mortality

## Abstract

**Background:**

Insecticide resistance in major malaria vectors poses severe challenges for stakeholders responsible for controlling the disease. During the 2013/14 season, malaria vector sentinel sites in Mutare and Mutasa Districts, Zimbabwe, experienced high presence of gravid malaria vector mosquitoes resting indoors in recently pyrethroid-sprayed structures. Subsequently, an evaluation of insecticide resistance in *Anopheles funestus* populations, the major malaria vector, was conducted to better inform the Zimbabwe National Malaria Control Programme.

**Methods:**

Indoor-resting mosquitoes were collected in randomly selected pyrethroid-sprayed houses around Burma Valley and Zindi sentinel sites in Mutare and Mutasa Districts, respectively, using prokopac aspirator in February 2014. *A. funestus* mosquitoes were identified in the field using morphological keys and divided into two cohorts. One cohort was used immediately for WHO susceptibility tests and the other batch was transferred to the National Institute of Health Research insectary in Harare for oviposition. Susceptibility and intensity resistance assays were carried out on polymerase chain reaction-assayed, 3–5 days old, *A. funestus s.s.* F1 progeny females.

**Results:**

Eight-hundred and thirty-six *A. funestus* and seven *Anopheles gambiae* complex mosquitoes were collected resting inside living structures. Wild caught females showed resistance to lambda-cyhalothrin (3.3 % mortality), deltamethrin (12.9 % mortality), etofenprox (9.2 % mortality), and bendiocarb (11.7 % mortality). F1 *A. funestus* female progeny indicated resistance to deltamethrin (14.5 % mortality), lambda-cyhalothrin (6.9 % mortality), etofenprox (8.3 % mortality), and bendiocarb (16.8 % mortality). Wild caught and female progeny were susceptible to DDT and pirimiphos-methyl (100 % mortality). Intensity resistance assay to bendiocarb was 100 % mortality, while deltamethrin, lambda-cyhalothrin, and etofenprox had increased knockdown times with mortalities ranging between 66.7 and 92.7 % after 24-h exposures.

**Conclusion:**

This study is the first report of pyrethroid and carbamate resistance in *A. funestus* populations from Burma Valley and Zindi areas and indicates a major threat to the gains made in malaria vector control in Zimbabwe. In view of the current extension and intensity of such resistance, there is urgent need to set up a periodic and systematic insecticide resistance-monitoring programme which will form the basis for guiding the selection of insecticides for indoor residual spraying and distribution of pyrethroid-treated mosquito nets.

## Background

Human malaria remains one of the most important public health challenges worldwide. In 2013, there were an estimated 198 million episodes of malaria and about 584,000 deaths globally [1]. Among the malaria-endemic countries in sub-Saharan Africa, malaria contributed 20–30 % of the outpatient attendance in Zimbabwe, with about 1.5 million cases occurring annually over the past 5 years [2]. Approximately 98 % of the cases are caused by *Plasmodium falciparum* transmitted primarily by *Anopheles arabiensis*, with *Anopheles gambiae* sensu stricto and *Anopheles funestus s.s.*, the secondary vectors in most regions of the country. Choi et al. [3] and Sande et al. [4] have reported *A. funestus* as the major vector of malaria in Mutare and Mutasa Districts of Manicaland Province in Zimbabwe.

Improved diagnostic testing and a wider availability of effective medicines to treat malaria, as well as to control vectors predominantly through the use of indoor residual spraying (IRS) and long lasting insecticidal nets (LLINs), are the global key interventions for interruption of malaria transmission [5]. Several studies have shown the efficacy of IRS and LLINs in reducing malaria incidence in almost all settings [6, 7].

Malaria control in Zimbabwe relies heavily on IRS and LLINs to target endophilic and endophagic vector mosquitoes, respectively. Presently, IRS and LLINs depend on the four most common, WHO-recommended, classes of insecticides: organochlorines, organophosphates, pyrethroids, and carbamates. Of these, pyrethroids account for the majority of IRS coverage worldwide and are at the moment used in treatment of all LLINs [8].

Since the 1940s, residual spraying with dichloro-diphenyl-trichloro-ethane (DDT) and more recently pyrethroids has been National Malaria Control Programme’s (NMCP) dominant/primary vector control practice in Zimbabwe. Mosquito nets traditionally played a much smaller role until the introduction of LLIN campaigns under the universal coverage goal over the past few years. When the LLIN distribution campaign began, there was no clear rationale for the balance of LLINs and IRS coverage in Zimbabwe as guided by WHO [5] recommendations. The high reliance on insecticide-based malaria control in public health, agriculture and at household levels has increased the selection pressure exerted by insecticides on malaria vectors [9]. The emergence and spread of insecticide resistance among malaria vectors has placed global control efforts at high risk.

Insecticide resistance is the ability of an insect population to survive exposure to the dosage of a given compound that is lethal to the majority of individuals of a susceptible lineage of the same species [9]. Malaria vectors are able to resist the action of insecticides due to various resistance mechanisms. Among these mechanisms: metabolic resistance, which occurs when endogenous, insecticide-detoxifying enzymes become more efficient in metabolizing the insecticide, preventing it from reaching its target in the nervous system, and target site resistance, which results from modification on the site of action in resistant strains of vectors, such that the insecticide no longer binds effectively, are the most important, although metabolic resistance is the most common [10].

Pyrethroid resistance, conferred by reduced target site sensitivity arising from a single point mutation in the sodium channel gene, at times referred to as knockdown resistance, has been confirmed in *A. gambiae s.s.* in West, Central and East Arica [11]. A study by Hunt et al. [12] documented insecticide resistance to permethrin, deltamethrin, bendiocarb, and propoxur in *A. funestus* populations collected in Likoma Island in Lake Malawi. Chanda et al. [13] reported DDT, lambda-cyhalothrin and deltamethrin resistance in *A. funestus* and *A. gambiae s.s.* collected in Zambia. *A. funestus* collected in Mozambique and Uganda showed resistance to bendiocarb, permethrin, deltamethrin, and lambda-cyhalothrin [14, 15]. In Kwazulu/Natal, South Africa, *A. funestus* was found to be resistant to both pyrethroids and carbamates [16].

Despite the long history of IRS in Zimbabwe, there have been few instances when resistance has been recorded [17]. *A. arabiensis* resistance to benzene hexachloride was recorded in Chiredzi District [18], one relating to DDT in Gokwe [19], and more recently pyrethroid resistance in Gokwe [20]. However, there are no major published studies on insecticide resistance in *A. funestus* in Zimbabwe. The first *A. funestus* resistance to deltamethrin, lambda-cyhalothrin and bendiocarb was reported by Choi et al. [3] in Mandeya ward, Mutasa District.

The lack of data on the status of insecticide resistance in *A. funestus*, the presence of this vector in recently pyrethroid-sprayed houses in villages around Burma Valley, Mutare District, Zimbabwe, and nearby Zindi area in Mutasa District, and high dependency on pyrethroid-based IRS and LLINs, is a cause for concern to the Zimbabwe NMCP. This study was aimed at assessing the insecticide resistance in *A. funestus* populations from Burma Valley and Zindi areas in Mutare and Mutasa Districts, respectively.

## Methods

### Study sites

**Fig. 1:**
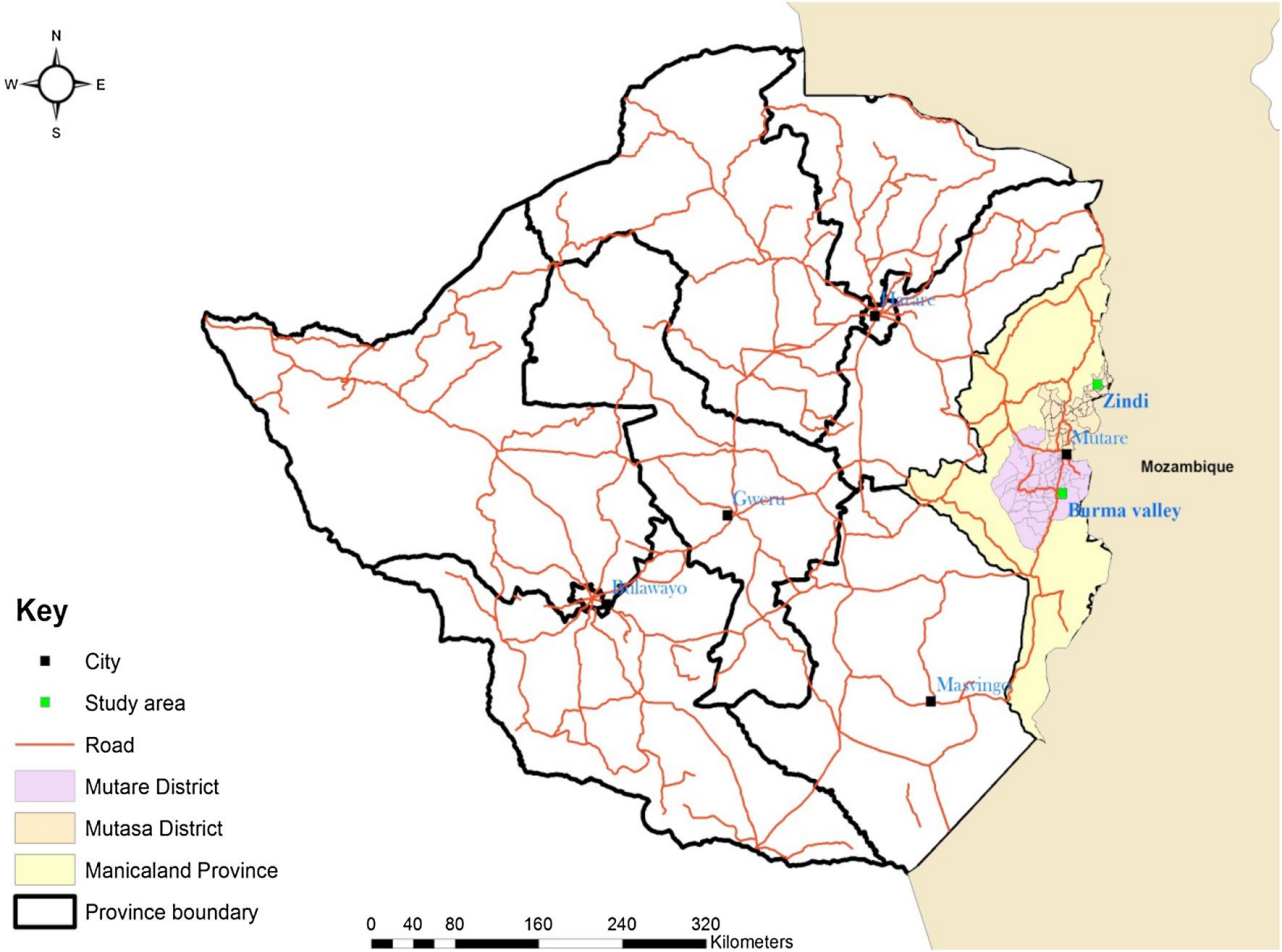
Map showing Burma Valley and Zindi study sites, Zimbabwe

The study was conducted in Mutare and Mutasa Districts in Manicaland Province, located east of Zimbabwe, 263 and 270 km, respectively, from Harare, and bordered to the east by Manica Province in Mozambique. The study sites were Burma Valley (19°11′S, 32°48′E; elevation 679 m) in Mutare District and Zindi (18°22′S, 32°56′E; elevation 766 m) in Mutasa District (Fig. 1). Burma Valley and Zindi sites are respectively situated south and north of the city of Mutare, the provincial capital of Manicaland Province. Studies were carried out from 10 to 23 February, 2014. Both study sites are rural areas with a total population of 13,880 (Burma Valley 4506 and Zindi 9374).

Domestic animals such as cattle, goats, chickens, and dogs are commonly kept around dwellings inhabited by people, with pigs found in only a few households. Two types of houses are common in the study sites: traditional houses, pole and mud plastered superstructures with thatched roofs, and western-style houses built using cement mortar and burnt bricks, roofed with either corrugated iron sheets or asbestos.

Both sites have a tropical climate which is hot, with annual temperature ranging from 18 to 30 °C in winter through to summer [21]. The rainfall pattern constitutes one season per annum which usually spans November to March, with December to February, the wettest months [21]. Most small rivers and streams in Zindi empty into perennial Pungwe River, which flows to Mozambique. Burma Valley, with several streams and perennial rivers, also runs to Mozambique. The rivers and streams form extensive stagnant water bodies and marshes during the rainy season (November–March), which are potential breeding sites for vectors of public health importance.

Cultivation on the river banks is common in the villages around the study sites. Both small and large-scale farming is practiced, with the majority of the people growing maize, yams and bananas. Eastern Highlands Estates located in Zindi ward, and a few small-scale commercial farms in Burma Valley, grow tea and tobacco, respectively. Both small and large-scale farmers usually use pyrethroids, organophosphates and carbamates to protect their crops from various types of agricultural pests. *A. funestus* is the major malaria vector in Burma Valley and Zindi [4], with the densities fluctuating following rainfall patterns, temperatures and relative humidity. Consequently, malaria transmission is high and occurs seasonally, with highest cases recorded towards the end of the rainy season (March/April). IRS and LLINs are the major tools deployed to interrupt malaria transmission in the villages around the study sites.

### Collection of *Anopheles funestus* populations

Indoor-resting adult mosquitoes were collected from houses between 06.00 and 10.00 hours using a prokopac battery-powered aspirator [22]. Live mosquitoes were identified to species level using morphological features [23, 24]. Mosquitoes identified as belonging to the *A. funestus* group were divided into two cohorts and held in cages where they were fed with 10 % sugar solution. One cohort was used immediately for WHO insecticide susceptibility bioassays and the other batch transferred to National Institute of Health Research (NIHR) insectary in Harare to allow for oviposition.

### Laboratory processing of mosquitoes

Live blood-fed and gravid adult female *A. funestus* were pooled and individually isolated and allowed to lay eggs. Larvae were reared through to F1 adults under standard insectary conditions of 25–27 °C and 70–80 % relative humidity. Polymerase chain reaction (PCR) using two legs per mosquito was carried out following the protocol of Koekemoer [25] to confirm the sibling species of all females that laid eggs, and susceptibility and intensity resistance assays were conducted on the F1 progeny only of *A. funestus s.s.* females.

### Insecticide susceptibility tests

Randomly selected, non-blood fed, F1 progeny (3–5 days old) and gravid wild caught samples were subjected to standard WHO susceptibility tests [26]. Standard insecticide-treated papers supplied by WHO (Malaysia) were used to test for susceptibility to 4 % DDT, 0.05 % deltamethrin, 0.05 % lambda-cyhalothrin, 0.5 % etofenprox, 0.1 % bendiocarb, and 1 % pirimiphos methyl. Twenty to 25 female mosquitoes were exposed in each tube. Negative controls consisted of untreated papers, impregnated with different oil according to the insecticide used. Knockdowns were recorded 10, 15, 20, 30, 40 min through to 1 h after the start of exposure. Final mortality was scored 24 h post exposure and a 10 % sugar solution was provided to survivors. Where the mortality in the control group was above 5 % but less than 20 %, correction of mortality was made by applying Abbott’s formula, with the test results discarded when control mortality was more than 20 %. Results were accepted if no mortality was observed in the control. WHO [26] criterion for interpretation of results was followed for considering vector species susceptible (mortality 98–100 %), potentially resistant (mortality 90–97 %) and resistant (mortality <90 %).

### Resistance intensity assays

F1 progeny female mosquitoes were exposed to 0.05 % lambda-cyhalothrin, 0.05 % deltamethrin, 0.5 % etofenprox, and 0.1 % bendiocarb-treated papers continuously for 8 h with knockdown being recorded at 5-, 10-, 15-, 20-, 30-, 40-, 50-, 60-, 80-, and 120-min intervals, and hourly thereafter up to 8 h. The 8-h cut-off was purposely selected as the likely time a mosquito might come into contact with a sprayed wall/surface before or after a taking blood meal [3].

### Data analysis

WHO [26] guideline for evaluating susceptibility in mosquito populations was followed in which mortality of 98–100 % indicates susceptibility; 90–97 % suggests potential resistance that needs to be confirmed, and less than 90 % indicates resistance. Data for the two study sites were tested using two-factor without replication Analysis of Variance (ANOVA), at 5 % level of significance.

### Ethical consideration

Verbal informed consent was obtained from community leaders and each head of household or representative before mosquito collection was conducted in the selected houses.

## Results

### Mosquito collection

A total of 846 *Anopheles* mosquitoes were collected resting inside recently pyrethroid-sprayed houses in the villages surrounding Burma Valley and Zindi over a two-week period in February 2014. Eight-hundred and thirty-six were identified morphologically as belonging to the *A. funestus* group, seven to the *A. gambiae s.l.* and the remaining three to other *Anopheles* species. Of the *A. funestus* group, 390 live mosquitoes were transported to NIHR for oviposition and PCR-based species identification, while 446 wild *A. funestus* female of unknown age were tested for insecticide resistance at the field insectaries with no temperature and relative humidity control. The results of these tests are summarized on Table 1. The wild-caught *A. funestus* group showed evidence of pyrethroid and carbamate resistance, but were susceptible to DDT and organophosphates. However, the sample size of wild *A. gambiae s.l.* females was too small (n = 7) to conduct meaningful susceptibility/resistance tests.

**Table 1:** Percentage mortality observed in WHO susceptibility tests carried out on wild caught members of the *A. funestus* group in Burma Valley and Zindi, Zimbabwe

Insecticide	Site	24 h post exposure		
		n	% mortality	Status
0.05 % lambda-cyhalothrin (pyrethroid)	Burma Valley	47	6.5	R
	Zindi	20	0	R
	*P* value (between sites)	–	0.35	–
0.05 % deltamethrin (pyrethroid)	Burma Valley	31	12.9	R
	Zindi	ND	–	–
	*P* value (between sites)	–	–	–
0.5 % etofenprox (pseudo-pyrethroid)	Burma Valley	33	3	R
	Zindi	39	15.4	R
	*P* value (between sites)	–	0.21	–
0.1 % bendiocarb (carbamate)	Burma Valley	38	21.1	R
	Zindi	43	2.3	R
	*P* value (between sites)	–	0.67	–
4 % DDT (organochlorine)	Burma Valley	36	100	S
	Zindi	30	100	S
	*P* value (between sites)	–	0.50	–
1 % pirimiphos-methyl (organophosphate)	Burma Valley	30	100	S
	Zindi	34	100	S
	*P* value (between sites)	–	0.50	–
Control	Burma Valley	35	0	–
	Zindi	30	0	–
	*P* value (between sites)	–	0.50	–

*ND* not done

### Mosquito rearing and PCR-species identification

From the 390 samples transported to NIHR insectary, 220 oviposition Eppendorf tubes were set up with individual gravid *A. funestus* females, about 134 batches were obtained, and more than 1900 F1 adult mosquitoes emerged from both sites. The results from the PCR-based assays confirmed that all the 220 females that laid eggs, as well as the 446 wild adults used in the susceptibility/resistance test, were *A. funestus s.s.*, while analysis of wild *A. gambiae s.l.* showed that *A. arabiensis* was predominant (71.4 %, 5/7) followed by a non-malaria vector, *Anopheles quadriannulatus* (28.6 %, 2/7).

### Insecticide susceptibility assays

Table 2 presents the mean mortalities and the standard deviations of *A. funestus* F1 progeny females that originated from the villages in Burma Valley and Zindi following exposure to insecticide-treated papers. Mortality in unexposed controls from both sites was less than 5 % in all experiments and no correction of test sample mortality data was therefore required. The treated papers used were assayed on a susceptible laboratory strain of *A. arabiensis* and showed 100 % mortality for all specimens and replicates (n = 100 mosquitoes per insecticide). *A. funestus* was resistant to lambda-cyhalothrin, deltamethrin and etofenprox (pyrethroids), and bendiocarb (carbamate), but susceptible to DDT (organochlorine) and pirimiphos-methyl (organophosphate) at both collecting sites. There was no significant difference in mortality of mosquitoes from Burma Valley and Zindi after exposure to pyrethroids (ANOVA: *df* = 4; F = 0.23; *P* = 0.92) and to bendiocarb (ANOVA: *df* = 1; F = 0.18; *P* = 0.71). The difference in percentage mortality between pyrethroid and carbamate assays and sites was also not statistically significant (ANOVA: *df* = 1; F = 4.39; *P* = 0.13).

**Table 2:** WHO bioassay tests for resistance on 3–5 day old female F1 *A. funestus* progeny from Burma Valley and Zindi carried out in February 2014

Insecticide	24 h % observed mortality			
	n (‡)	% mortality (range)	Standard deviation	Resistance status
0.05 % lambda-cyhalothrin	100 (4)	9 (4–13.8)	3.5	R
0.05 % deltamethrin	87 (4)	12.6 (10.8–14.7)	1.5	R
0.5 % etofenprox	90 (4)	3.3 (1.6–4.9)	1.3	R
0.1 % bendiocarb	98 (4)	25.5 (21.3–28.8)	2.8	R
4 % DDT	100 (4)	100	0	S
1.0 % pirimiphos methyl	100 (4)	100	0	S
Untreated control	129 (5)	0.8 (0–1.8)	0.9	–
0.05 % lambda-cyhalothrin	107 (5)	4.7 (3.8–5.7)	0.6	R
0.05 % deltamethrin	92 (4)	16.3 (14.0–18.4)	1.6	R
0.5 % etofenprox	83 (4)	13.3 (11.8–14.9)	1.1	R
0.1 % bendiocarb	100 (4)	8 (5.5–10.2)	2	R
4 % DDT	114 (5)	100	0	S
1.0 % pirimiphos methyl	96 (4)	100	0	S
Untreated control	122 (5)	0	0	–

‡, number of tubes/replicates; R, resistant; S, susceptible

### Knockdown effect of insecticide on F1 *Anopheles funestus* progeny females

Common similarities were observed in KD_50_ and KD_95_ values between the pyrethroids (lambda-cyhalothrin and deltamethrin) and carbamates (bendiocarb), and between organochlorines (DDT) and organophosphates (pirimiphos-methyl) in both Burma Valley and Zindi sites (Table 3). The knockdown effects of the four classes of insecticides tested over 1 h showed more rapid knockdown rate for DDT and pirimiphos-methyl than the other two classes of insecticides (Table 3). DDT knocked down 50 and 95 % of the mosquitoes from both sites within 50 and 60 min of exposure, respectively. Fifty per cent and 95 % knockdown was achieved within 50 and 80 min, respectively, for mosquitoes collected from Zindi when exposed to pirimiphos-methyl. There was loss of knockdown effect on all samples from both sites when exposed for 80 min to lambda-cyhalothrin and deltamethrin and bendiocarb.

**Table 3:** Association between percentage 24-h mortality and knockdown (KD) time using WHO test tubes

Insecticide	Site	% mortality	KD_50_ (min)	KD_95_ (min)	Resistance status
0.05 % lambda-cyhalothrin	Burma Valley	9.0	No KD	No KD	R
	Zindi	4.7	No KD	No KD	R
0.05 % Deltamethrin	Burma Valley	12.6	No KD	No KD	R
	Zindi	16.3	No KD	No KD	R
0.1 % Bendiocarb	Burma Valley	25.5	No KD	No KD	R
	Zindi	8.0	No KD	No KD	R
0.5 % Etofenprox	Burma Valley	3.3	No KD	No KD	R
	Zindi	13.3	No KD	No KD	R
4 % DDT	Burma Valley	100	50	60	S
	Zindi	100	40	50	S
1 % Pirimiphos-methyl	Burma Valley	100	30	60	S
	Zindi	100	50	80	S

S, susceptible; R, resistant; KD, knockdown; KD_50_, knockdown rate for 50 % of mosquitoes; KD_95_, knockdown rate for 95 % of mosquitoes; No KD, loss of knockdown effect (<20 % of mosquitoes knocked down after 1-h exposure)

### Insecticide resistance intensity in *Anopheles funestus* F1 female progeny

**Fig. 2:**
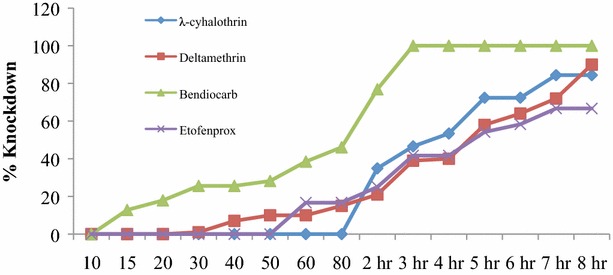
Insecticide intensity resistance test in *Anopheles funestus* in Burma Valley

**Fig. 3:**
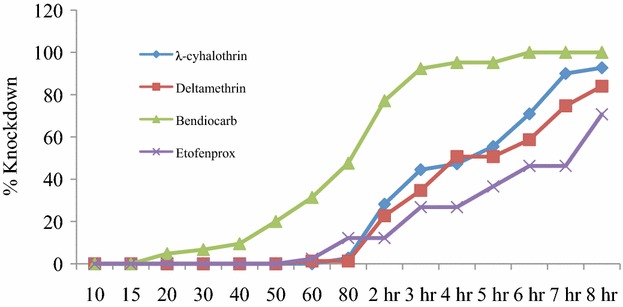
Insecticide intensity resistance test in *A. funestus* in Zindi

*Anopheles funestus* exhibited various levels of knockdown effects after 8-h exposure to insecticides, with highest sensitivity observed in bendiocarb for the two localities (Table 4). In both areas, there was no statistically significant difference in responses among lambda-cyhalothrin, deltamethrin and etofenprox over the entire 8-h observation period (ANOVA: *df* = 5; F = 2.39; *P* = 0.11). Although knockdown rate for deltamethrin in Burma Valley and Zindi sites were observed from 30 and 80 min, respectively, the sensitivity of the mosquitoes to the insecticide could not stretch beyond 90 % knockdown effect within an 8-h monitoring period (Figs. 2, 3). Similarly, observations on lambda-cyhalothrin and etofenprox showed percentage knockdown rate of less than 100 % for the entire experimental period in both sites.

**Table 4:** Resistance intensity results of F1 progeny raised from female *A. funestus* collected in Burma Valley and Zindi

Insecticide	Location	n (§)	KD_50_ (min)	% knockdown after 8-h exposure (range)	Standard deviation
0.05 % λ-cyhalothrin	Burma Valley	58 (3)	240	84.4 (83.7–85.4)	0.7
	Zindi	110 (5)	300	92.7 (90.2–94.2)	1.7
0.05 % deltamethrin	Burma Valley	100 (4)	300	90 (86.2–93.7)	2.7
	Zindi	75 (3)	240	84 (80.9–86.6)	2.4
0.1 % bendiocarb	Burma Valley	39 (2)	120	100	0
	Zindi	105 (5)	80	100	0
0.5 % etofenprox	Burma Valley	24 (1)	300	66.7	–
	Zindi	41 (2)	480	70.7 (69.8–71.6)	0.9

§, number of tubes/replicates

## Discussion

The status of susceptibility/resistance to lambda-cyhalothrin, deltamethrin, etofenprox, bendiocarb, DDT and pirimiphos-methyl was evaluated in *A. funestus* wild populations and F1 progeny females collected from Burma Valley and Zindi in Zimbabwe. With guidance from WHO [26] protocol for characterizing insecticide resistance, where susceptibility is defined by mortality rates above 98 % and resistance by mortality less than 90 % 24-h post exposure, this study provides evidence that *A. funestus* populations from both sites were resistant to pyrethroids and carbamates, but susceptible to DDT and pirimiphos-methyl. The information is important in malaria vector control operations, which in Zimbabwe are strongly dependent on the use of insecticides in IRS and LLINs.

Zimbabwe has been using DDT for both tsetse fly and malaria vector control since 1949 [20]. Currently, DDT is being applied for malaria vector control in the low veld zones of Zimbabwe (<600 m altitude), and pyrethroids, especially lambda-cyhalothrin and deltamethrin, are used interchangeably to cover the middle veld zones (600–1200 m altitude). Continuous application of DDT and alternating lambda-cyhalothrin with deltamethrin might increase selection pressure, resulting in early loss of sensitivity in vector populations. Choi et al. [3] reported *A. funestus* resistance to lambda-cyhalothrin, deltamethrin and bendiocarb for the first time in the area adjacent to the Zindi collecting site, but no DDT and organophosphate-resistant populations were detected in that area. These findings are consistent with the results of the present work, which showed resistance in *A. funestus* to pyrethroids and bendiocarb. In both areas, there was no statistically significant difference in 24-h mortality among lambda-cyhalothrin, deltamethrin, etofenprox, and bendiocarb (ANOVA: *df* = 7; F = 0.93; *P* = 0.51). The detection of deltamethrin and lambda-cyhalothrin resistance in the *A. funestus* populations in Burma Valley and Zindi is a worrying result as these are the most common insecticides applied interchangeably by NMCP in Zimbabwe to prevent malaria transmission in the study areas.

The build-up of pyrethroid and carbamate resistance in the *A. funestus* populations from the two study areas is not clear. Most probably the increase in the selection pressure exerted by pyrethroids may be attributed to their continuous use in public health, agriculture and at household level to control domestic pests. Bendiocarb resistance may be mainly associated with application in agriculture, which is a major source of livelihood in both study areas. The incrimination of agricultural use of pesticides in the selection pressure against *Anopheles* populations has also been reported in several countries in West Africa [27, 28]. Since pyrethroid resistance has been reported to result mainly from agricultural application, it is likely that such resistance will develop regardless of the organized use of pyrethroids in properly managed malaria control programmes [29].

Results of the present work agree with other studies that reported pyrethroid and bendiocarb resistance in the *A. funestus* populations from Malawi [12], Zambia and Zimbabwe [3], Mozambique [30], and Ghana [31]. The reported occurrence of permethrin and DDT resistance in malaria vectors in Gokwe District in Zimbabwe [20] was not detected in *A. gambiae s.l.* populations from 16 sentinel sites (Burma Valley and Zindi included) in Zimbabwe following a nationwide study [17]. Resistance to pyrethroids generally confers cross-resistance to other insecticides with the same mode of action, thus limiting the alternative choices of effective insecticide [32]. The lack of cross-resistance between pyrethroids and DDT observed in this study is consistent with the work of Coetzee and Koekemoer [33], which reported that pyrethroid resistance in *A. funestus* is mostly conferred fully or partially by monooxygenases (P450) in most countries in southern Africa. Further, it appears there is no knockdown resistance *(kdr)* gene in southern African *A. funestus* to date [33], as is also clearly indicated by the observation of this work. However, cross-resistance to pyrethroids and DDT has been reported in most mosquito species of public health importance collected from other countries as a result of a *kdr* gene [34, 35].

In addition to mortality, knockdown time might be a valuable tool for the early detection of reduced susceptibility, although there are no WHO standards on knockdown time specified to indicate resistance. Knockdown time has long been accepted as an indicator of susceptibility in vector mosquitoes to insecticides. The time provides initial data on the possible involvement of *kdr* gene [29], although high frequency of a resistant gene does not necessarily translate into resistance in *Anopheles* populations [36].

The results of this work have demonstrated elevated knockdown time for all insecticides tested, with the increase more pronounced in pyrethroids and carbamates than DDT and organophosphate. However, there was no difference between the time required to knockdown 100 % of the mosquitoes due to DDT and pirimiphos-methyl from the two sites (ANOVA: *df* = 3; F = 1.91; *P* = 0.23). Although this study detected no resistance to DDT and pirimiphos-methyl, the KD_50_ and KD_95_ values obtained from both sites for these two insecticides appear to be abnormally high, ranging from 30 to 80 min to knockdown 100 % of the specimens. These results may be an indication of future problems with the application of DDT and pirimiphos-methyl in Burma Valley and Zindi.

The high survival rates and the increased knockdown time detected in this study raise the question of whether mosquitoes are withstanding higher concentrations of insecticide or whether longer exposure times are needed. To address the latter question, a resistance intensity test was included in the current study in order to determine the strength of resistance, although it is not the standard method of measuring resistance. Currently, the standard methods of anopheline bioassays are the WHO [26] tube assay and the CDC [37] bottle assay. Although the two methods generally agree on resistance frequencies, there has been no agreement on the application of resistance intensity test as a standard tool for measuring insecticide resistance in mosquito populations.

The CDC [37] bottle bioassay method recommends the extension of diagnostic time to 2 h in order to evaluate intensity of resistance, but does not give criteria for assessing resistance intensity. Within 2 h of continuous exposure, *A. funestus* from both sites showed mortality of less than 40 % to lambda-cyhalothrin, deltamethrin and etofenprox, with about 80 % to bendiocarb, suggesting a high level of resistance to the pyrethroids. A problem with not achieving 100 % knockdown after an 8-h exposure time to all insecticides used, save for bendiocarb, might indicate serious resistance intensity. At operational level, this poses a major challenge as it is not clear whether a mosquito rests continuously for 8 h on a sprayed surface or on a treated net, taking into cognisance the repellency and irritancy properties contained in various insecticides.

## Conclusion

Focusing on the pattern emerging from the two study sites, it is clear that *A. funestus* resistance to pyrethroids and carbamates, and susceptibility to DDT and pirimiphos-methyl, are firmly indicated. The resistance in the *A. funestus* populations detected in this study has serious implications for the current insecticide-based malaria control efforts being undertaken in the study areas. The results seem to suggest the need for urgent and effective insecticide resistance management strategies necessary for the prevention of rapid build-up of resistance across all four commonly used classes of insecticides to control vectors of public health importance.
